# Targeting of mutant p53-induced FoxM1 with thiostrepton induces cytotoxicity and enhances carboplatin sensitivity in cancer cells

**DOI:** 10.18632/oncotarget.2497

**Published:** 2014-12-04

**Authors:** Xuan Zhang, Lihua Cheng, Kay Minn, Rashna Madan, Andrew K. Godwin, Viji Shridhar, Jeremy Chien

**Affiliations:** ^1^ Department of Cancer Biology, University of Kansas Medical Center, Kansas City, Kansas, U.S.A; ^2^ Department of Pathology and Laboratory Medicine, University of Kansas Medical Center, Kansas City, Kansas, U.S.A; ^3^ Department of Laboratory Medicine and Pathology, Mayo Clinic, Rochester, Minnesota, U.S.A

## Abstract

FoxM1 is an oncogenic Forkhead transcription factor that is overexpressed in ovarian cancer. However, the mechanisms by which FoxM1 is deregulated in ovarian cancer and the extent to which FoxM1 can be targeted in ovarian cancer have not been reported previously. In this study, we showed that MDM2 inhibitor Nutlin-3 upregulated p53 protein and downregulated FoxM1 expression in several cancer cell lines with wild type *TP53* but not in cell lines with mutant *TP53*. FoxM1 downregulation was partially blocked by cycloheximide or actinomycin D, and pulse-chase studies indicate Nutlin-3 enhances FoxM1 mRNA decay. Knockdown of p53 using shRNAs abrogated the FoxM1 downregulation by Nutlin-3, indicating a p53-dependent mechanism. FoxM1 inhibitor, thiostrepton, induces apoptosis in cancer cell lines and enhances sensitivity to cisplatin in these cells. Thiostrepton downregulates FoxM1 expression in several cancer cell lines and enhances sensitivity to carboplatin *in vivo*. Finally, FoxM1 expression is elevated in nearly all (48/49) ovarian tumors, indicating that thiostrepton target gene is highly expressed in ovarian cancer. In summary, the present study provides novel evidence that both amorphic and neomorphic mutations in TP53 contribute to FoxM1 overexpression and that FoxM1 may be targeted for therapeutic benefits in cancers.

## INTRODUCTION

Forkhead Box M1 (FoxM1), a member of the Forkhead family of transcription factors, is overexpressed in the majority of human cancers. It has been found to play an important role in cancer development by regulating multiple biological processes such as cell proliferation, differentiation, survival, and migration [1]. A genome-wide study reported that FoxM1 mRNA was overexpressed in most high-grade serous ovarian carcinomas without DNA copy number changes [2].

Three isoforms of FoxM1 have been identified to date. FoxM1A, which harbors all of the ten exons of FoxM1 gene, is transcriptionally inactive. FoxM1B lacks exons Va and VIIa, while FoxM1C possesses Va but lacks VIIa; both are transcriptionally active [3]. FoxM1 has been implicated in cell cycle control [4–7], proliferation [8–11], DNA damage signaling [12, 13], invasion, angiogenesis, metastasis [11, 14–16], resistance to cancer drugs [13, 17–20], and aggressive tumor behavior and clinical outcomes [14–16, 21, 22]. FoxM1 activity is regulated at the expression level by growth factors [18, 23], and at the post-translational level by phosphorylation which enhances its nuclear localization and nuclear transcriptional activities [24]. At the transcriptional control level, FoxM1 expression is regulated by Sp1 and KLF4 [16], E2F [13], FoxO3 [20], HIF-1 [25], and c-Myc [8, 26]. In addition, p53 has been shown to repress FoxM1 expression [12, 13].

*TP53* encodes for a tumor suppressor that mediates cell-cycle arrest, apoptosis, and/or cellular senescence by either stimulating or repressing down-stream target genes [27]. The importance of p53 in cancer surveillance and therapeutics has been well studied [28]. Loss of p53 activity inhibits apoptosis and accelerates the appearance of tumors in transgenic mice [29]. *TP53* missense mutations can inactivate not only the normal function, but also exert pro-oncogenic effects [30]. Mutations in *TP53* are common in human cancers [31], and approximately 95% of high-grade serous ovarian cancer harbor *TP53* mutations [2]. Although two studies investigated the potential role of p53 in the regulation of FoxM1 expression [12, 13], these studies focused on transcriptional regulation via E2F or FoxO3. In addition, the results from these studies are inconsistent, for example Barsotti & Prives [12] reported that FoxM1 downregulation by p53 is dependent on p21 whereas Millour *et al* [13] did not find p21-dependent repression of FoxM1 by p53, suggesting the need to improve our understanding of the mechanisms regulating FoxM1 expression by p53 in cancers.

Considering that *TP53* mutations and FoxM1 overexpression occur in most ovarian cancer, we were intrigued to explore the regulation of FoxM1 by p53 in ovarian cancer cells. p53 protein is tightly regulated by MDM2, an E3 ubiquitin ligase that ubiquitinates p53 and promotes p53 degradation [32]. Nutlin-3 is a small molecule that inhibits p53 degradation by interacting with the p53-binding pocket of MDM2 and suppressing p53-MDM2 interaction [33]. *TP53* null cells showed minimal changes genome-wide expression following Nutlin-3 treatment, indicating that Nutlin-3 is selective for p53 [34, 35]. Therefore, in this study we investigated the mechanisms of FoxM1 regulation by p53 in cancer cell lines using Nutlin-3 as a tool.

## RESULTS

### Nutlin-3 upregulates p53 and downregulates FoxM1 protein in cancer cells with wild type *TP53*

**Figure 1:**
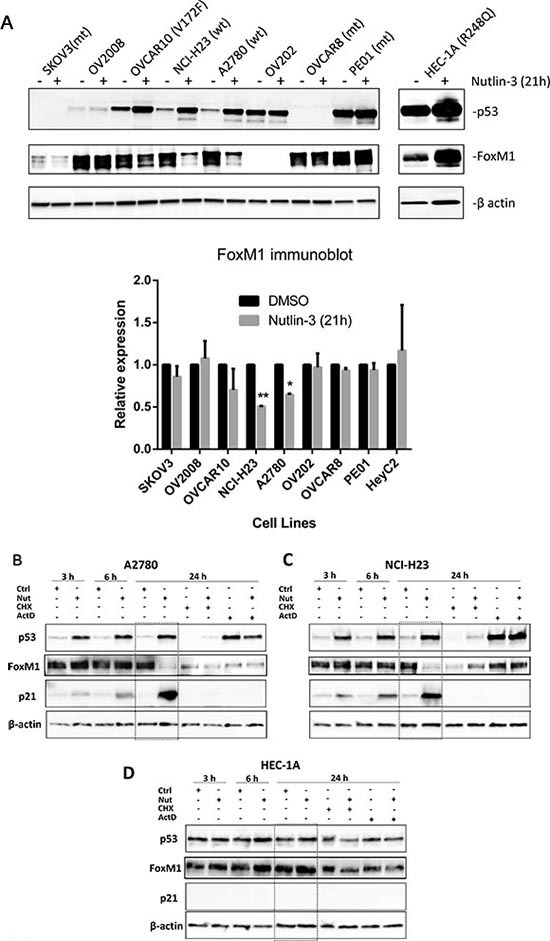
Functional p53 is required for FoxM1 suppression by Nutlin-3 **(A)** In cell lines with functional p53, p53 expression is induced, and FoxM1 expression is suppressed by Nutlin-3. Cell lines with known *TP53* mutations are indicated by “mt” and wild type cell lines are indicated by “wt”. Note, although OVCAR10 contains a mutant allele (V172F), p53 expression is induced, and FoxM1 expression is suppressed by Nutlin-3, suggesting the wild type copy is sufficient to suppress FoxM1 expression. Significant down-regulation of FoxM1 was observed in NCI-H23 (**, P ≤ 0.01) and A2780 (*, P ≤ 0.05). FoxM1 expression was normalized with β-actin and was expressed relative to DMSO-treated controls. **(B-D)** Time-course experiments indicate that p53 is induced within 3 hours of Nutlin-3 treatment in A2780 and NCI-H23 cells with wild type p53, and FoxM1 downregulation is observed at 24 hours in these cells. In HEC-1A cells with mutant p53, neither p53 nor FoxM1 was affected by Nutlin-3 at various time points. These results indicate functional p53 is required for FoxM1 downregulation by Nutlin-3. Cells were treated with vehicle (0.05% DMSO), 10 μM Nutlin-3 for 3, 6 or 24 h, or with CHX, CHX+Nutlin-3, ActD or ActD+Nutlin-3 for 24 h. Proteins were isolated and subjected to Western analysis. β-actin was used for normalization of loading. Downregulation of FoxM1 at 24 hours following Nutlin-3 treatment is highlighted in the dotted box.

To begin to understand the regulation of FoxM1 by p53 in cancer cells, we first examined the effects of MDM2 inhibitor Nutlin-3 on the expression of p53 and FoxM1 proteins in several cancer cell lines with either wild-type or mutant *TP53* (Figure 1A). We observed that Nutlin-3 treatment for 21h resulted in an increase in p53 protein levels in OVCAR10, NCI-H23 and A2780 cells that have functional *TP53*, but not in cell lines with known *TP53* mutations (SKOV3 [36], OVCAR8 [37], and PE01 [38], HEC-1A[39]) and nor in cell lines (OV2008, OV202) where p53 dysfunction was suspected (Figure 1A). Variable basal expression of FoxM1 protein was detected in all cell lines tested, and a decrease in FoxM1 levels was observed in association with p53 upregulation by Nutlin-3. FoxM1 levels remained unchanged in *TP53* mutant cell lines and in OV2008 and OV202 cell lines that failed to respond to Nutlin-3. These results suggest that FoxM1 suppression by Nutlin-3 may be partly dependent on functional p53.

### Downregulation of FoxM1 protein by Nutlin-3 is dependent on functional p53 and is attenuated by cycloheximide and actinomycin D

To explore the mechanisms for Nutlin-3-induced downregulation of FoxM1 in cancer cells with functional p53, we first examined the time-course of FoxM1 protein expression in A2780 and NCI-H23 and its association with p53 and p21, a well-known p53 transcription target. A *TP53* mutant cell line HEC-1A was included as a control. As shown in Figure 1B&C, an increase in p53 and p21 protein levels was observed as early as 3 h post treatment in A2780 and NCI-H23 cells and became more dramatic by 24 h, consistent with the functional status of p53. FoxM1 levels, however, were not decreased until 24 h. We then included a protein synthesis inhibitor cycloheximide (CHX) and a transcription inhibitor actinomycin D (ActD), alone or in combination with Nutlin-3, in 24 h treatment groups to help decipher the mechanism. CHX treatment alone for 24 h decreased p53 protein expression in A2780 and NCI-H23 cells as compared to controls. CHX + Nutlin-3 combination treatment resulted in minimal increased p53 protein levels as compared to CHX alone, indicating that Nutlin-3 increases p53 protein stability, which is in agreement with the literature [35, 40]. FoxM1 protein levels in these cells were decreased by CHX treatment as well. Co-treatment with Nutlin-3 did not lead to further decrease in FoxM1 protein levels, indicating that downregulation of FoxM1 by Nutlin-3 is not due to decreased protein stability.

Interestingly, ActD, alone or in combination with Nutlin-3, was able to increase p53 protein levels in A2780 and NCI-H23 cells without a marked decrease in FoxM1 protein expression levels, suggesting the possibility that FoxM1 downregulation requires *de novo* transcription. Upregulation of p53 protein expression in ActD-treated cells is not unexpected because p53 expression is regulated at the post-transcriptional level by MDM2-mediated ubiquitination and degradation [41, 42]. We also observed that FoxM1 protein levels were lower in Nutlin-3-treated group as compared to CHX + Nutlin-3 or ActD + Nutlin-3 groups in both A2780 and NCI-H23 cells, indicating that downregulation of FoxM1 protein by Nutlin-3 can be partially blocked by either CHX or ActD. p21 protein levels became undetectable following treatment with CHX or ActD in A2780 and NCI-H23 cells, further corroborating the inhibitory effect of CHX and ActD on *de novo* translation and transcription, respectively. Nutlin-3 did not significantly alter p53 or FoxM1 protein expression in HEC-1A cells with mutant *TP53* (Figure 1D). p21 protein expression was undetectable in HEC-1A cells throughout treatment. These results further suggest the role of functional p53 in FoxM1 suppression.

### Downregulation of FoxM1 mRNA by Nutlin-3 is dependent on functional p53 and is blocked by cycloheximide and actinomycin D

**Figure 2:**
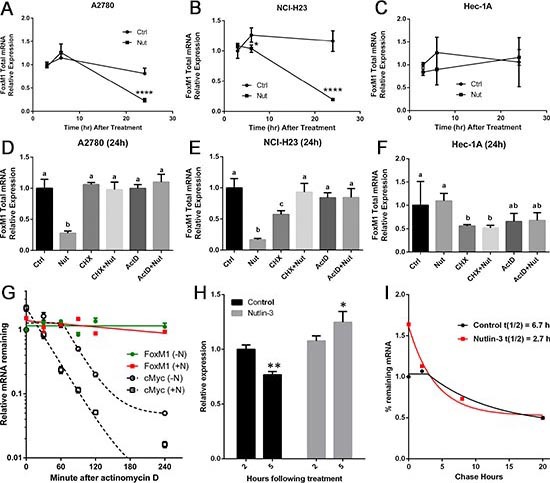
FoxM1 mRNA is downregulated by Nutlin-3 in *TP53* wild type cells but not in *TP53* mutant cells Upper panel **(A-C)**: Cells were treated with vehicle (0.05% DMSO) or 10 μM Nutlin-3 for 3, 6 or 24 h. Lower panel **(D-F)**: Cells were treated with DMSO, Nutlin-3, CHX, CHX+Nutlin-3, ActD or ActD+Nutlin-3 for 24 h. Total RNA was isolated and subjected to real-time RT-PCR analysis. GAPDH was used for normalization of FoxM1 expression. It is important to note that FoxM1 mRNA is quite stable in the presence of ActD in cell lines with wild type *TP53* **(D&E)**. Data are presented as Mean ± SD of 3 experiments. **** indicates P < 0.0001 by paired t-test. Difference alphabet letters indicate significant differences (P < 0.05) across treatments by One-Way ANOVA. Nutlin-3 treatment enhances FoxM1 mRNA decay. **(G)** Analysis of the effect of Nutlin-3 on FoxM1 mRNA stability using actinomycin D. A2780 cells were treated with or without Nutlin-3 for 14 hours, followed by actinomycin D treatment for 30, 60, 120, or 240 min. Total RNA was isolated and subjected to real-time RT-PCR analysis of FoxM1. cMyc mRNA was used as a positive control. **(H)** Effects of Nutlin-3 on nascent FoxM1 transcription in A2780 cells. Click-iT EU labeling followed by real-time RT-PCR was used to compare nascent FoxM1 mRNA synthesis at 2 h or 5 h after treatment with vehicle or Nutlin-3. * indicates P < 0.05; ** indicates P < 0.01. **(I)** Effects of Nutlin-3 on FoxM1 mRNA decay. A2780 cells were treated with or without Nutlin-3 for 5 h before pulse-labeling for 1 h. Total RNA was isolated at 0, 2, 8, or 20 h post labeling and subjected to RT-PCR analysis. FoxM1 mRNA half-life was calculated using GraphPad Prism6.

To examine the extent to which FoxM1 suppression by Nutlin-3 is attributed to decreased FoxM1 steady-state mRNA levels and to investigate the mechanism of regulation, A2780, NCI-H23, and HEC-1A cells were treated with or without Nutlin-3 for 3, 6, or 24 h, and FoxM1 expression was analyzed using real-time RT-PCR. In accordance with the Western data, FoxM1 mRNA levels remained unaltered at 3 h and 6 h, but were significantly decreased by 24 h in both cell lines with functional p53 (Figure 2A&B). In HEC-1A cells, FoxM1 mRNA levels did not significantly change across the time points following Nutlin-3 treatment (Figure 2C).

Further analysis showed that downregulation of FoxM1 mRNA levels by Nutlin-3 could be completely blocked by either CHX or ActD in both A2780 and NCI-H23 cells, suggesting that *de novo* protein synthesis was involved in the downregulation of FoxM1 by Nutlin-3 (Figure 2D&E). ActD alone for 24 h did not lead to a significant decrease in FoxM1 mRNA levels, which suggests that FoxM1 mRNA is relatively stable, as least in the presence of ActD in cells with wild type p53 (Figure 2D&E). Interestingly, in HEC-1A cells with mutant p53, ActD significantly downregulates FoxM1 mRNA levels (Figure 2F), suggesting that mutant p53 may have different effect on FoxM1 expression. We also examined the expression of three FoxM1 isoforms separately (Figure S1–S3), and observed results similar to total FoxM1 expression.

### Nutlin-3 reduces FoxM1 mRNA stability

To test if Nutlin-3 treatment alters FoxM1 mRNA stability, we quantified FoxM1 total mRNA levels in cells treated with or without Nutlin-3 for 14 hours, followed by ActD treatment for variable duration. cMyc mRNA was used as a positive control. We observed a rapid decay of cMyc mRNA following the transcription inhibition by ActD. The half-life of cMyc mRNA was estimated to be approximately 40 minutes (Figure 2G). In contrast, the half-life of FoxM1 mRNA in cells treated with Nutlin-3 was estimated to be approximately 400 minutes while the half-life of FoxM1 mRNA in cells treated with DMSO was unavailable due to lack of mRNA decay within the test time frame. These results further support our hypothesis that Nutlin-3 enhances FoxM1 mRNA decay.

Because we observed that FoxM1 mRNA downregulation was partially blocked by actinomycin D, suggesting that *de novo* transcription is required, we were concerned that actinomycin D treatment during FoxM1 mRNA decay analysis may cause artificial stability of FoxM1 mRNA and may produce spurious half-life data. We, therefore, applied an alternative method of Click-It EU labeling that does not require ActD treatment to determine the decay. Moreover, this method can also be used to determine nascent mRNA synthesis after Nutlin-3 treatment. We first determined the extent to which Nutlin-3 treatment affected nascent FoxM1 mRNA synthesis. We treated A2780 cells with Nutlin-3 for either 2 hours or 5 hours, followed by 1 hour pulse labeling with Click-It EU nucleotide. We then performed Click-It chemistry to biotinylate nascent mRNA that were subsequently purified through streptavidin beads. Quantitative RT-PCR analysis of nascent mRNAs from these samples indicates that Nutlin-3 treatment did not inhibit FoxM1 transcription (Figure 2H). In fact, we observed an increase in FoxM1 transcription at 5 hours of Nutlin-3 treatment.

We next determined the FoxM1 mRNA decay rate in A2780 cells pretreated for 5 hours with Nutlin-3, followed by 1 hour pulse-labeling with Click-IT EU, and chased for various time-points after washout. Quantitative RT-PCR analysis indicates fast rate of FoxM1 mRNA decay in cells treated with Nutlin-3 (t_½_ = 2.7 hours) compared to cells treated with DMSO (t_½_ = 6.7 hours) (Figure 2I). It should be noted that the half-life of FoxM1 mRNA in Nutlin-3 treated cells with ActD chase was estimated as approximately 6.5 hours and differed from the estimate obtained from Click-IT EU label and chase experiment. The difference is likely due to ActD blocking *de novo* transcription of secondary factor(s) that regulates FoxM1 mRNA stability.

### Knock-down of p53 by shRNAs blocks Nutlin-3-induced FoxM1 downregulation

**Figure 3:**
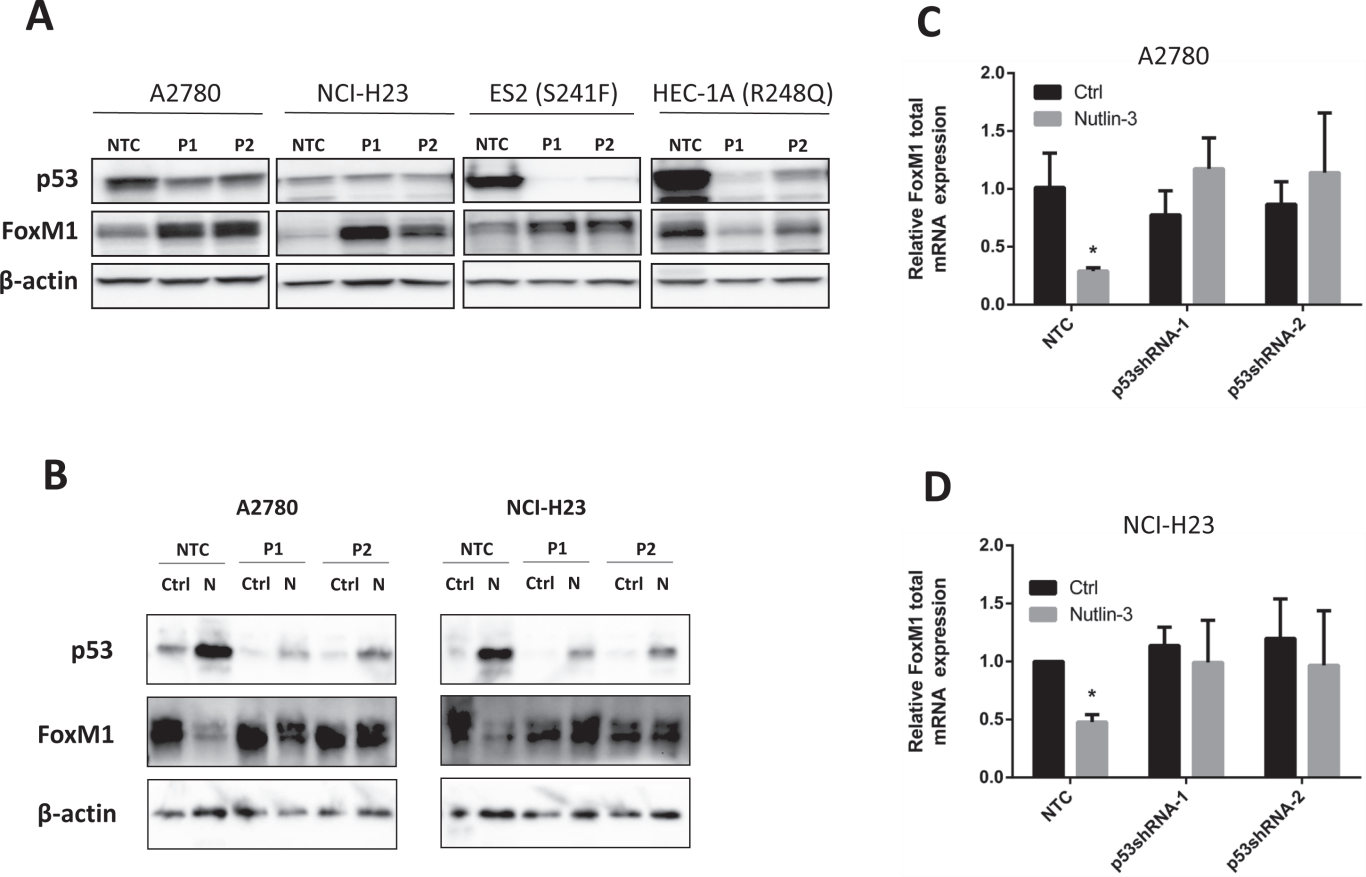
Endogenous wild type p53 suppresses FoxM1 expression whereas R248Q mutant p53 enhances FoxM1 expression **(A)** Effects of p53 knockdown using shRNAs on FoxM1 protein expression in p53 WT A2780, NCI-H23, and p53 mutant HEC-1A and ES2 cell lines. Endogenous FoxM1 is upregulated when wild type TP53 is knocked down by two shRNAs in A2780 and NCI-H23 cells. Similarly, downregulation of S241F mutant TP53 also upregulate FoxM1 expression, suggesting that S241F mutant retains negative regulatory effect on FoxM1 expression. In contrast, downregulation of R248Q in HEC-1A cells downregulates FoxM1 expression, suggesting that R248Q mutant may have gain of positive regulatory effect of FoxM1 expression. Proteins were isolated from p53 shRNA batch clones and subjected to Western analysis. β-actin was used for normalization of loading. **(B)** p53 knockdown using shRNAs blocks the downregulation of FoxM1 protein expression by Nutlin-3 in A2780 and NCI-H23 cells. Cells were treated with or without Nultin-3 for 24 h. Proteins were then isolated and subjected to Western analysis. β-actin was used for normalization of loading. **(C-D)** p53 knockdown blocks the downregulation of FoxM1 mRNA by Nutlin-3 in A2780 and NCI-H23 cells. Cells were treated with or without Nultin-3 for 24 h. Total RNA was isolated and subjected to real-time RT-PCR analysis. GAPDH was used for normalization of FoxM1 expression. Data are presented as Mean ± SD of 3 experiments. * indicates P < 0.05 by paired t-test.

We then designed experiments to confirm that downregulation of FoxM1 by Nutlin-3 was dependent on the upregulation of functional p53. To do this, we generated batches of stable cell lines each expressing two different p53-targeting shRNAs in A2780, NCI-H23, ES2, and HEC-1A cells. Cells stably expressing non-targeting control shRNA (NTC) served as the control. As shown in Figure 3A, knockdown of p53 protein expression by p53 shRNA-1 (P1) or p53 shRNA-2 (P2) resulted in increased expression of FoxM1 protein in both A2780 and NCI-H23 cells as compared to NTC. To our surprise, knockdown of p53 in *TP53* mutant HEC-1A cells resulted in a decreased in FoxM1 protein levels, suggesting the possibility that mutant *TP53* in HEC-1A positively regulates FoxM1 expression. In contrast, the knockdown of S241F mutant in ES2 cells resulted in upregulation of FoxM1. These results highlight the heterogeneity in the regulation of FoxM1 by p53 mutants, with S241F mutant still retaining the negative regulatory effect on FoxM1 expression while TP53 mutant R248Q acquires the gain of positive regulatory effect on FoxM1 expression.

Next, we treated the A2780 and NCI-H23 cells, expressing NTC or p53-targeting shRNAs, with or without Nutlin-3 for 24 h and analyzed the expression of p53 and FoxM1 proteins by Western blots. As expected, knockdown of p53 attenuated p53 induction by Nutlin-3 and partially blocked the effects of FoxM1 downregulation (Figure 3B). To confirm that this is attributed to regulation at the mRNA level, cells with the same treatment were subjected to real-time RT PCR analysis. Consistent with the Western data, knockdown of p53 in A2780 and NCI-H23 cells blocked the downregulation of FoxM1 mRNA levels by Nutlin-3 (Figure 3C&D), indicating that functional p53 is necessary for Nutlin-3-induced FoxM1 downregulation.

### FoxM1 inhibitor, thiostrepton, downregulates FoxM1 expression and induces apoptosis in cancer cell lines

**Figure 4:**
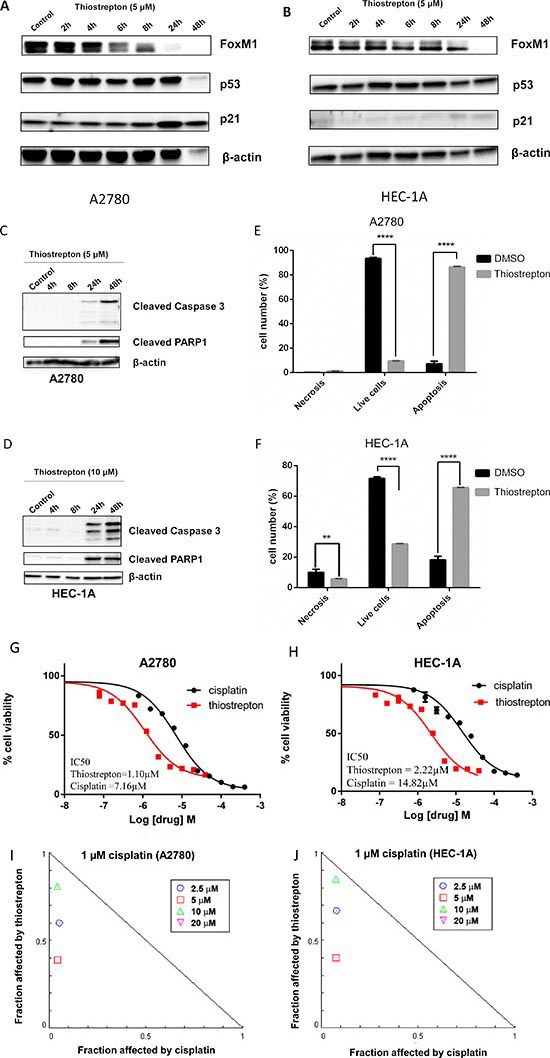
FoxM1 inhibitor thiostrepton downregulates FoxM1 expression and induces cytotoxicity in cancer cell lines with wild type or mutant *TP53* **(A-B)** Thiostrepton downregulates FoxM1 expression in A2780 (A) and HEC-1A (B). **(C-D)** Thiostrepton treatment results in caspase-3 and PARP1 cleavage in cancer cells. **(E-F)** Quantification of early and late apoptosis by flow cytometry with Annexin-V and propidium iodide (PI) staining indicates that thiostrepton induces apoptosis in cancer cell lines. **(G-H)** Thiostrepton suppresses cell viability in A2780 (G) and HEC-1A (H). Cytotoxicity induced by cisplatin (circle) was used as a comparison. The IC_50_ for thiostrepton was estimated to be 1.10 μM in A2780 and 2.22 μM in HEC-1A compared to 7.16 μM (A2780) and 14.82 μM (HEC-1A) for cisplatin. **(I-J)** Lower concentrations of thiostrepton (2.5, 5, and 10 μM) show synergistic drug interactions with 1 μM cisplatin in both A2780 (G) and HEC-1A (H) cell lines. 20 μM thiostrepton shows antagonistic interaction with cisplatin, and is not shown in the graph. Normalized isobolograms were calculated using CompuSyn. Drug effects shown below the diagonal additivity line signify synergistic drug interactions.

To assess the potential of FoxM1 as a therapeutic target, we treated gynecologic cancer cell lines with FoxM1 inhibitor, thiostrepton. Previous studies indicated that thiostrepton inhibits FoxM1 transcription factor activity and consequently downregulates FoxM1 expression [43–45]. Consistent with these results, we observed downregulation of FoxM1 by thiostrepton in several ovarian cancer cell lines (Figure 4A&B and Figure S4). Thiostrepton induces apoptosis in these cell lines as evidenced by the cleavage of caspase-3 and PARP1 (Figure 4C & 4D). Since we observed apoptotic morphology in A2780 and HEC-1A within 24 h and 48 h respectively (data not shown), we used these time points to quantify apoptosis in these cells using Annexin V labeling. The results, shown in Figure 4E&F and Figure S5A&B, indicate cancer cells underwent apoptosis following thiostrepton treatment.

### Thiostrepton suppresses cell viability and enhances sensitivity to cisplatin

To assess the therapeutic potential and synergistic interactions with cisplatin, we treated A2780 and HEC-1A with various concentrations of thiostrepton and cisplatin, separately, as well as together at various ratios of drug concentrations. Thiostrepton induces apoptosis as evidenced by Annexin V labeling and cell viability in both *TP53* wild type and mutant cell lines. The IC_50_ for thiostrepton was estimated to be 1.10 μM in A2780 and 2.22 μM in HEC-1A compared to 7.16 μM (A2780) and 14.82 μM (HEC-1A) for cisplatin (Figure 4G&H). In addition, at lower concentrations of thiostrepton (2.5, 5, and 10 μM), we observed synergistic drug interactions with 1 μM cisplatin in both A2780 and HEC-1A cell lines (Figure 4I&J).

### FoxM1 enhances carboplatin sensitivity *in vivo*

**Figure 5:**
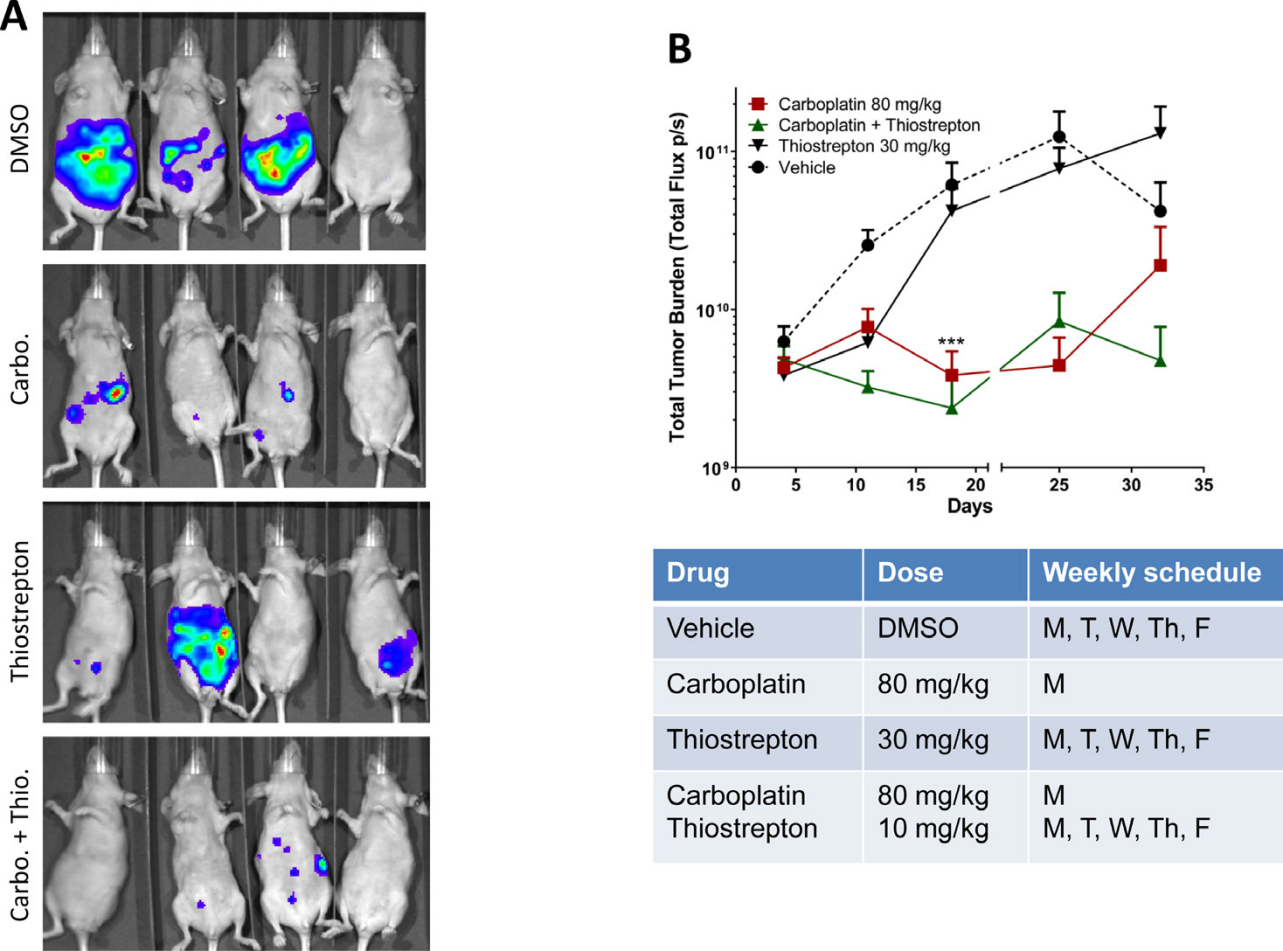
Thiostrepton enhances in vivo carboplatin sensitivity in HEC-1A cancer cells **(A)** Luciferase-label HEC-1A (2.5 million cells/mouse) cells were intra-peritoneally injected into nude mice, and in vivo bioluminescence imaging was perform 1 week later. Mice were placed into four groups (7-9 mice per group) (DMSO, carboplatin, thiostrepton, and carboplatin plus thiostrepton) and treated with corresponding drugs. Weekly bioluminescence imaging was performed to monitor tumor growth. Representative images taken at Day 25 are shown. **(B)** Total photon flux were collected, and mean values plus standard errors were plotted as line graphs. A significant decrease in tumor volume was observed at Day 18 (week 3) in the groups treated with carboplatin alone or in combination with thiostrepton. ***, p < 0.001 in multiple t-test using Holm-Sidak method at α = 0.05. The graph was plotted as two sections with a break at Day 21 to indicate change in dosing of carboplatin.

To assess *in vivo* therapeutic potential of thiostrepton, we treated mice bearing HEC-1A tumor xenografts with carboplatin alone, thiostrepton alone, or in combination. DMSO (vehicle) treatment served as a control group. A weekly dose of 80 mg/kg of carboplatin was selected based on previous studies indicating that up to 120 mg/kg weekly dose can be given in mice [46]. Representative bioluminescence images of tumor growth at Day 25 are shown in Figure 5A. We observed a significantly measurable response at Day 18 in the groups treated with carboplatin alone or in combination with thiostrepton compared to DMSO group at corresponding time point (Figures 5B). However, we also observed the evidence of toxicity from carboplatin treatment as indicated by a drastic weight loss in mice treated with 80 mg/kg carboplatin (Figure S6), and therefore the dose was reduced to 20 mg/kg starting at Day 22. As a result of suboptimal carboplatin dose, we observed a trend suggestive of tumor progression in the group of mice treated with carboplatin alone (Figure 5B). Interestingly, despite the suboptimal dose of carboplatin, the group of mice treated with the combination of carboplatin and thiostrepton did not show evidence of tumor progression (Figure 5B). However, as a result of large variations in bioluminescence imaging and tumor necrosis in DMSO group (Figure S7), we did not observe a significant difference in tumor burden at Day 25 and 32. These results, although limited by technical challenges with *in vivo* imaging and variable growth dynamics, support the potential preclinical activity of the carboplatin and thiostrepton combination.

### FoxM1 is overexpressed in ovarian carcinomas

**Figure 6:**
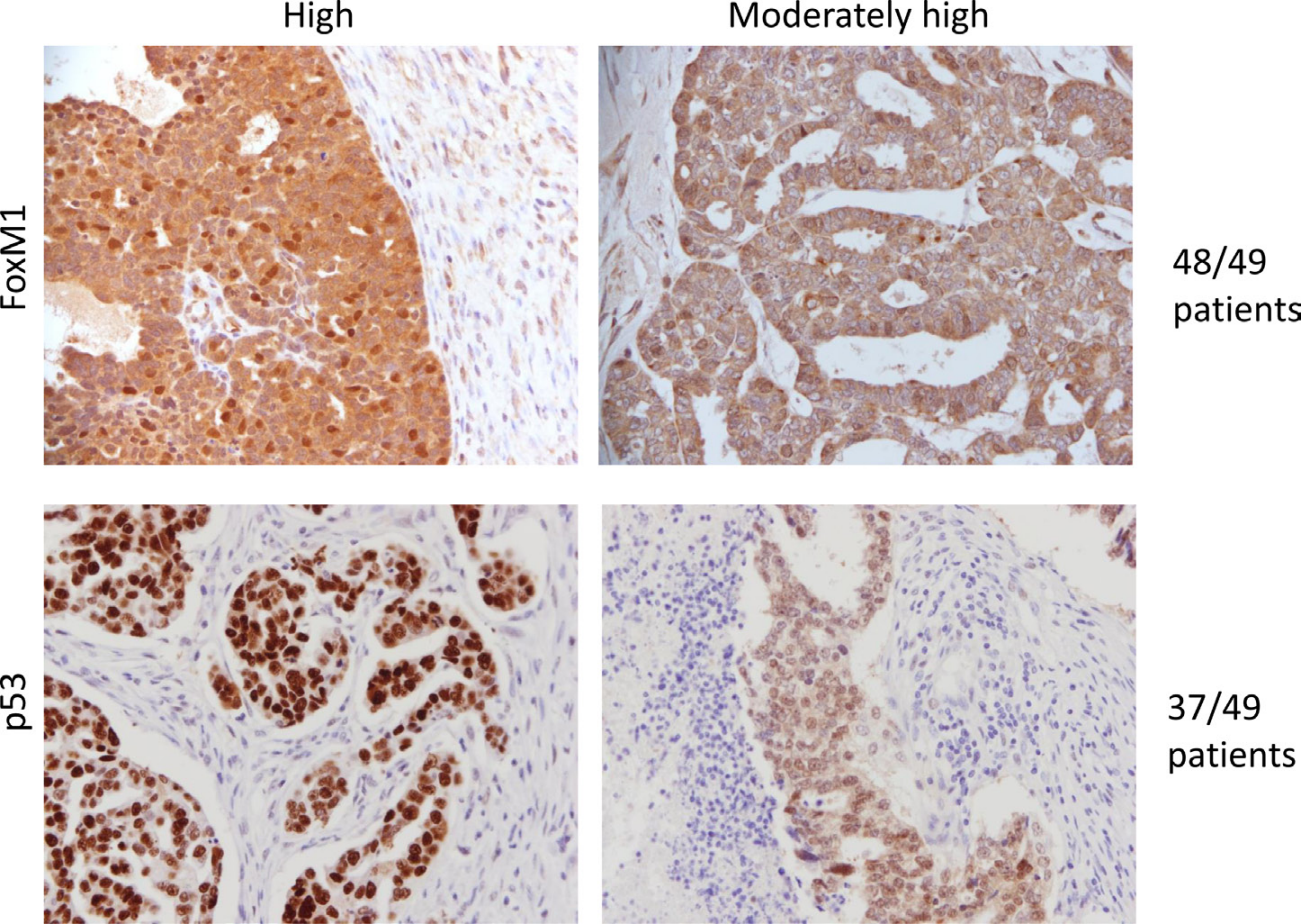
FoxM1 expression is elevated in tumor tissue Tumor tissue microarray (TMA) was used to analyze the expression of FoxM1 in ovarian tumor samples. 48 out of 49 tumors show high or moderately high levels of FoxM1 expression, and stromal compartment shows minimal expression of FoxM1. In comparison, 37 out of 49 tumors showed high or intermediate levels of p53.

Finally, to determine the extent to which the target (FoxM1) is expressed in ovarian carcinomas, we performed immunohistochemistry on ovarian tumor tissue microarray (Supplemental Table 1). Results indicate that 48 out of 49 tumors have moderate to high levels of FoxM1 expression (Figure 6). Moreover, FoxM1 expression is higher in tumor cells than surrounding stroma (Figure 6). We also observed intense but diffuse staining of p53 in 76% (37/49) carcinoma samples, suggestive of harboring somatic mutations in *TP53*. In the remaining tumors without diffuse p53 staining, we observed complete loss of p53 staining, again reflectively of somatic mutations in *TP53* resulting in a null phenotype.

## DISCUSSION

Previous studies reported that p53 negatively regulates FoxM1 expression through transcriptional repression and implicated p21 and E2F as potential negative and positive regulator of FoxM1 expression [12, 13]. In this study, we uncovered another level of regulation on FoxM1 expression by p53, which is mediated through regulation of FoxM1 mRNA stability. First, our results indicate that FoxM1 is downregulated by Nutlin-3 in A2780 and NCI-H23 cancer cells, and this regulation is dependent on functional p53. In addition, this suppression requires *de novo* transcription and translation because cycloheximide and actinomycin D attenuated Nutlin 3-mediated suppression of FoxM1 expression. Consistent with the role of functional p53 in FoxM1 suppression, downregulation of p53 by RNAi not only attenuated Nutlin 3-mediated suppression on FoxM1 expression but also induced basal FoxM1 levels in p53 knockdown cells with wild type *TP53*.

Interestingly, downregulation of mutant p53 by RNAi in HEC-1A cells reduced basal FoxM1 expression levels, suggesting that mutant p53 positively regulates FoxM1 expression whereas wild type p53 negatively regulates FoxM1 expression. This may partially explain the deregulated FoxM1 expression in various cancer as well as in ovarian cancer because *TP53* is frequently mutated in human carcinomas [2, 47]. This finding is not completely unexpected because research in recent years has clearly shown that p53 gain-of-function mutations can function as a pro-oncogenic factor and induce distinct changes in gene expression [30, 48]. We are excited to report this novel observation although it was not the original focus of the experimental design in this study. On-going studies in our laboratory are continuing to investigate the role of oncogenic *TP53* mutations in the regulation of FoxM1 expression.

In this study, the effect of p53 on FoxM1 total, FoxM1A, FoxM1B, and FoxM1C are almost identical, indicating that downregulation of FoxM1 by p53 is not isoform-specific. The exact function of each isoforms in ovarian cancer is unknown at this time. Based on studies in other cancer models, FoxM1A may be transcriptionally inactive, while FoxM1B and FoxM1C are transcriptionally active and regulate oncogenic phenotypes [49].

It is interesting to note that FoxM1 mRNA levels were not immediately suppressed by Nutlin-3 although p53 was rapidly induced by Nutlin-3. The relatively slow kinetics of FoxM1 mRNA suppression suggests the involvement of secondary factors regulating FoxM1 expression. The *de novo* synthesis of one or more of these factors is necessary in this process. One possible explanation is that p53 may stimulate the expression of certain miRNA through mechanisms involving *de novo* synthesis. This miRNA then binds to FoxM1 mRNA and induces its degradation. However, we have not yet identified any miRNA that mediates this effect. In fact, we have examined the expression of miRNA 134, which is known to target FoxM1 [50] but failed to observe an induction by Nutlin-3 (data not shown). *In silico* analysis of miRTarBase [51] indicates three other miRNAs (miR-26b, 186, and 149) as potential regulator of FoxM1 expression. Further studies are needed to identify the factors involved in decreasing FoxM1 mRNA stability by p53 in this study.

**Figure 7:**
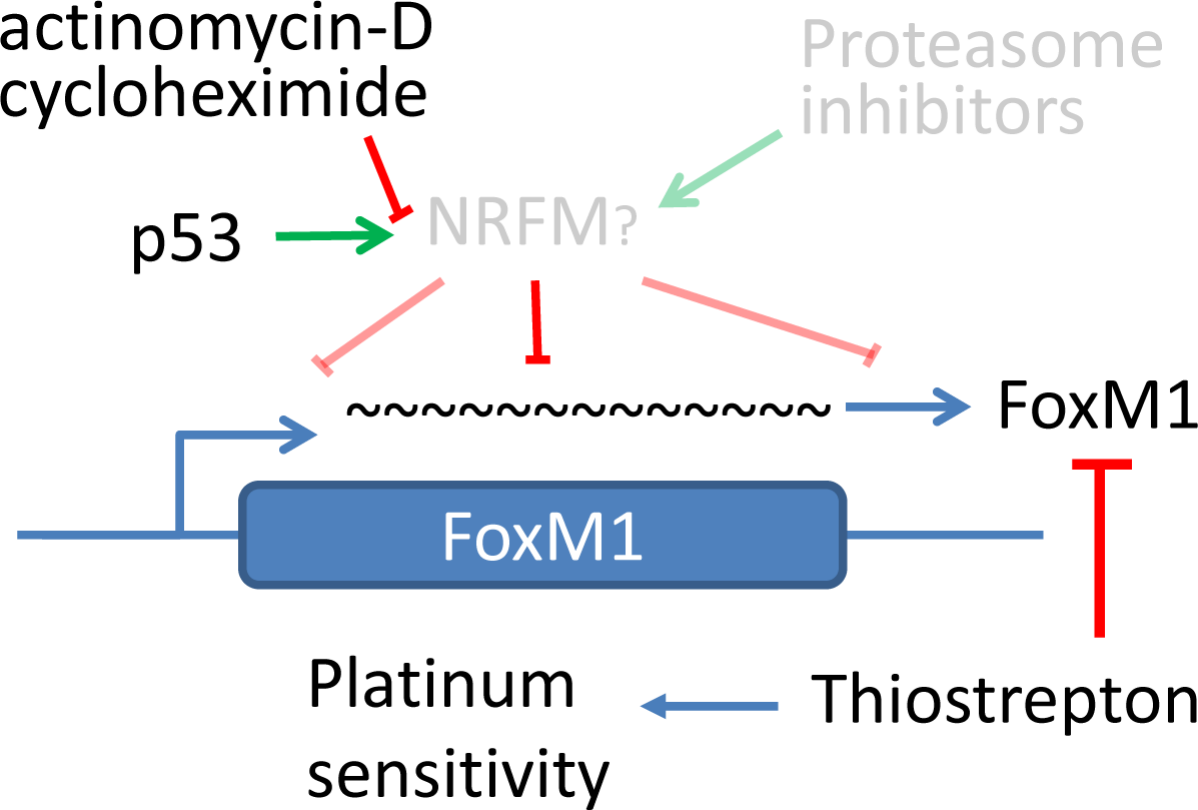
Hypothetical model of FoxM1 regulation by p53 through a putative negative regulator FoxM1 is negatively regulated by a putative negative regulator of FoxM1 (NRFM) as previously proposed by Andrei Gartel. Putative NRFM may regulate FoxM1 expression at protein level by enhancing FoxM1 degradation or via mechanisms involving decreased mRNA stability. p53 stimulates the expression/function of this NRFM through transcription- and translation-dependent mechanisms, and consequently downregulates FoxM1. Transcription inhibitor actinomycin D or translation inhibitor cycloheximide blocks FoxM1 downregulation by p53. Prior model, shown in gray, is proposed by Gartel et al [53], and indicates that putative NRFM may be stabilized by proteasome inhibitors. Such putative NRFM with high rate of turnover may be stabilized by proteasome inhibitors but may be destabilized by inhibition of constitutive expression by either ActD or CHX, leading to enhanced FoxM1 stability.

Previous studies utilizing proteasome inhibitors indicate that these inhibitors suppress FoxM1 expression [52], and it is proposed that the stabilization of a hypothetical negative regulator of FoxM1 (NRFM) by proteasome inhibitors may account for the suppression of FoxM1 expression [53]. Here, our results provide an alternative to the role of the hypothetical NRFM by providing evidence that *de novo* transcription and translation is required to downregulate FoxM1 mRNA and that post-transcriptional regulation of FoxM1 mRNA stability also contributes to FoxM1 downregulation (Figure 7). These results are consistent with the proposed model of a putative NRFM with a high rate of turnover. Such putative factor would be upregulated by proteasome inhibitors leading to FoxM1 downregulation, but it would be downregulated by ActD or CHX leading to FoxM1 stability.

Targeting FoxM1 pathway with FoxM1 inhibitor thiostrepton induces apoptosis as evidenced by increased Annexin-V labeling and caspase-3 and PARP1 cleavage. Thiostrepton also suppresses cell viability as determined by Alamar Blue staining in TP53 wild type as well as mutant cells. Cytotoxicity induced by thiostrepton was more potent than that induced by cisplatin in these cells. Consistent with previous reports in other cancer cell lines, thiostrepton downregulates FoxM1 expression in several ovarian cancer cell lines as well as in endometrial (HEC-1A) and lung (NCI-H23) cancer cell lines. Finally, thiostrepton enhances sensitivity to cisplatin *in vitro* and carboplatin *in vivo*. It is important to note that we do not observe anti-tumor activity of thiostrepton at 30 mg/kg dose that was tested in this study. The limitations of our studies are that we evaluated just one dose of thiostrepton as a single agent and that bioavailability, pharmacokinetics, and pharmacodynamics of thiostrepton were not investigated. Moreover, as a result of aggressive tumor growth in DMSO-treated mice and consequence necrosis, we observed large variations tumor burden in this study. Therefore, in future studies, it is therefore important to investigate several doses of thiostrepton as well as bioavailability, pharmacokinetic and pharmacodynamics of thiostrepton in ovarian xenograft models. In addition, it would be important to investigate the *in vivo* anti-tumor activity of another FoxM1 inhibitor, Siomycin A, and its potential synergistic activity with carboplatin in ovarian tumor xenograft and patient-derived xenograft models. Finally, it will be important to pre-determine tumor growth kinetics so that drug response is measured during the growth phase of tumor to avoid confounding factors resulting from tumor necrosis.

Off-target effect of thiostrepton is a concern. Prior studies have shown that thiostrepton directly interacts with FoxM1 and inhibits the binding of FoxM1 to target genes [44]. However, interactions between thiostrepton and other cellular targets cannot be excluded. Nonetheless, its ability to downregulate FoxM1 expression and induce cytotoxicity in p53 mutant as well as wild type cancer cell lines is significant because it could potentially allow us to target a component of TP53 gain-of-function and loss-of-function effect.

In summary, we identified the post-transcriptional regulation of FoxM1 mRNA stability as a novel mechanism by which FoxM1 expression is regulated by wild type p53. We showed that p53, induced by Nutlin-3, enhances FoxM1 mRNA decay, and this effect requires *de novo* transcription and translation. In addition, we found that mutant p53 can positively regulate FoxM1 expression. These observations point to two potential mechanisms of FoxM1 upregulation in ovarian cancer: Both the loss of wild type TP53 and the gain of oncogenic mutant TP53 may contribute to FoxM1 overexpression. In particular, we found that R248Q mutation of TP53 in HEC-1A positively regulate FoxM1 expression, and this observation represents a novel gain-of-function phenotype of R248Q mutant. R248Q mutant has been previously described as a neomorphic mutation because R248Q/− mice showed accelerated tumor onset and death, and R248Q/+ Li-Fraumeni patients also have accelerated tumor onset [54]. Since metastatic behavior and tumor progression have been attributed to both FoxM1 and R248Q mutation in TP53 [16, 54–56], in future studies it would be important to delineate the role of FoxM1 in tumor progression associated with the R248Q mutation in TP53. In addition, our studies identify FoxM1 as a potential therapeutic target in several cancer types. These discoveries are expected to advance our understanding of p53-FoxM1 axis in cancer and may ultimately allow rational targeting of this pathway for therapeutic purposes.

## MATERIAL AND METHODS

### Cell lines and reagents

Cancer cell lines including SKOV3, OV2008, A2780, NCI-H23, OVCAR10, HEC-1A, ES2, PE01, OVCAR 8, and OVCAR3 were maintained in MCDB105 and M199 (1:1) containing 5% FBS, 100 units/Ml penicillin and 100 μg/mL streptomycin. The antibodies against FoxM1 and β-actin were purchased from Sigma (St. Louis, MO). The antibodies against p53 and p21 were purchased from Santa Cruz (Dallas, Texas). Nutlin-3, cycloheximide (CHX), and actinomycin D (ActD) were purchased from Sigma. Final concentrations for Nutlin-3, CHX, and ActD were 10 μM [57], 25 μg/ml [58], and 5 μg/ml [59], respectively.

### Western analysis

Cells were collected at the end of treatments, and total proteins were extracted using radioimmuno-precipitation assay (RIPA) buffer containing a protease/phosphatase inhibitor cocktail (Cell signaling). Protein concentrations were determined using the BCA protein assay reagent kit (Pierce, Rockford, IL). Equal amount of proteins were subjected to SDS-polyacrylamide gel electrophoresis and electroblotted onto PVDF membranes. After blocking with 5% nonfat dry milk in TBS-Tween for 2 h at room temperature, blots were incubated with appropriate primary antibodies overnight at 4°C. Blots were then washed and incubated with appropriate horseradish peroxidase (HRP)-conjugated secondary antibodies for 1 h and protein bands were visualized using a chemiluminescence kit (Thermo Scientific, Rockford, IL). Next, blots were stripped and re-probed for β-actin. The expression level of each protein was normalized to the level of β-actin.

### Real-time quantitative RT-PCR (qPCR)

Total RNA was extracted from cancer cells using Trizol reagent. cDNA was synthesized using iScript Reverse Transcription Supermix (Bio-Rad). The resulting cDNA was diluted 1:5 in sterile water, and 1 μl aliquots was used in the qPCR reactions. Primers were designed with Primer3 plus. qPCR was carried out on a CFX384 Real-Time System (Bio-Rad). A no-template reaction was included during each experiment to control for DNA contamination in the reagents. Amplification of GAPDH was used to normalize the level of mRNA expression. Each cDNA sample was run in triplicate. Primers used in the assays are shown in Supplemental Table 2.

### Click-iT Nascent RNA Capture and Real-Time RT-PCR analysis

A Click-iT Nascent RNA Capture kit (Invitrogen) was used to examine FoxM1 mRNA transcription and stability according to the protocol provided by the manufacturer. Briefly, nascent RNA in cancer cells was pulse-labeled with 5-ethynyl uridine for 1 h. Total RNA was isolated at various time points after labeling and washout and used in a copper catalyzed click reaction with an azide-modified biotin. Biotinylated RNA was captured on streptavidin magnetic beads and reverse transcribed into cDNA, which was used in the real-time RT-PCR analysis. For analysis of FoxM1 nascent mRNA transcription, cells were treated with DMSO or 10 μM Nutlin-3 for 2 h or 5 h before pulse-labeling and immediate isolation of RNA. For FoxM1 mRNA stability analysis, cells were treated with vehicle or 10 μM Nutlin-3 for 5 h followed by 1 h pulse-labeling and washout. Cells were then chased for 0, 2, 4 or 20 h before RNA isolation.

### Stable short hairpin RNA-mediated downregulation of p53 and FoxM1

GIPZ p53 or FoxM1 lentiviral shRNAs (Thermo Scientific) were transfected into 293T cells using Trans-Lentiviral Packaging Kits (Thermo Scientific). Supernatants containing lentiviral particles were collected 48–64 h post-transfection. The transductions were carried out in A2780, NCI-H23 or HEC-1A cells 24 h after seeding. Batch stable clones were selected by puromycin. The efficiency of knockdown was determined by Western blot analysis. shRNAs used in the studies are shown in Supplemental Table 3.

### AlamarBlue cytotoxicity assay

Cells were plated into 96-well plates (2 × 10^3^cells / 200 μl/well) and cultured in growth medium overnight. The next day, cells were treated with various concentrations cisplatin, thiostrepton or in combinations of both drugs. 48 hours later, cells viability was assessed using alamarBlue® (Life Technologies). The IC50 for each drug was determined by GraphPad Prism (version 6) using dose-response function. Isobologram for drug synergies was determined using open source program CompuSyn (http://www.combosyn.com/) [60].

### *In vivo* efficacy study utilizing the hec-1a intraperitoneal tumor model

Athymic Nu/Nu female mice (7–9 week old) were inoculated with luciferase expressing HEC-1A cells (2 × 10^6^) by intraperitoneal (i.p.) injection. Briefly, exponentially growing HEC-1A-luc cells were harvested and injected into peritoneal cavity of mice using a 27 gauge needle. Three days after tumor inoculation, mice were imaged using the IVIS Spectrum optical imaging unit and baseline tumor burden determined for each mouse. To normalize the tumor burden for each group, mice that lack tumor take, as determined by bioluminescence imaging, were dropped from each group, resulting in 7 to 9 mice per group for the study. On day 4, treatment was initiated and mice were imaged once weekly for the duration of the study to determine the anti-tumor response to treatment. In two of the treatment groups, mice were administered either carboplatin (80 mg/kg) weekly or Thiostrepton (30 mg/kg) daily five days per week for monotherapy treatment. A third group received a combination of Carboplatin (80 mg/kg) and thiostrepton (10 mg/kg) dosed weekly and five days per week, respectively. The fourth group was a control group that received the vehicle for carboplatin (water) and thiostrepton (DMSO) using the combination therapy dosing schedule. After the first 3 weeks of drug-treatment the dose of Carboplatin was reduced to 20 mg/kg for the duration of the study. All animal studies were carried out in the animal facilities of The University of Kansas Medical Center with strict adherence to the guidelines of the IACUC Animal Welfare Committee of KUMC (IACUC approval # 2012–2067).

### *In vivo* imaging

Mice (7 to 9 mice for each group) were imaged on a weekly basis using IVIS spectrum imager. Briefly, animals were injected with potassium salt of D-Luciferin (15 mg/ml at 10 μl/gm bodyweight) followed by isoflurane induced anesthesia. Images were quantified using Living Image software version 4.0. Region of interest (ROI) boxes were drawn around the entire body of the animals. Measurements were expressed as flux, i.e. photons/second (p/s). Graphpad Prism (ver 6) was used to analyze the significance of differences in tumor response using the Two-way ANOVA and the Dunnett's multiple comparisons test.

### Statistical analysis

All data were analyzed using GraphPad Prism (version 6). Multiple t-tests were used for comparison between control and Nutlin-3 groups. ANOVA was used for comparison across treatment regimes. Significance was set at P < 0.05 for all comparisons.

### Tissue microarrays and immunohistochemistry

Tissue microarrays (TMAs) were constructed from archival formalin fixed, paraffin embedded samples of ovarian carcinoma from 48 patients. The TMAs also included matched metastases and recurrences from 14 of the aforementioned 48 patients (1 of these 14 patients had a late recurrences in the brain), matched metastases alone for 27 patients and matched recurrences alone for 7 patients. Using the semi-automated TMArrayer (Pathology Devices, Inc., Westminster, MD), TMA blocks were assembled with triplicate 1.0 mm cores of each tumor sample. Based on review of the original pathology reports, the ovarian carcinomas were typed as serous (30 samples), mixed (14 samples; 12 of which included a serous component), carcinosarcoma (1 sample), clear cell (1 sample), papillary carcinoma, not otherwise specified (NOS) (1 sample) and adenocarcinoma, NOS (1 sample).

Rabbit polyclonal antibody FOXM1(C-20) (Sc:502) from Santa Cruz Biotechnology, Inc. (Santa Cruz, CA) is used for immunohistochemical staining according to the following procedure. Four micron paraffin sections are mounted on Fisherbrand Superfrost* slides and baked for 60 minutes at 60′C then deparaffinized. Epitope retrieval was performed in Biocare Decloaking Chamber (pressure cooker), under pressure for 5 min, using pH 6.0 citrate buffer, followed by a 10 minute cool down period. Endogenous peroxidase is blocked with 3% H2O2 for 10 minutes followed by incubation with FOXM1 (1:200) primary antibody for 45 min., followed by Mach 2 HRP Polymer (Biocare Medical, Concord CA) for 30 minutes and DAB+ chromogen (Dako, Carpinteria, CA) for 5 minutes. Immunohistochemical staining was performed using the IntelliPATH FLX Automated Stainer at room temperature. A light hematoxylin counterstain was performed, following which the slides were dehydrated, cleared, and mounted using permanent mounting media.

## SUPPLEMENTARY FIGURES AND TABLES


