# Supplementary material for: Targeting of mutant p53-induced FoxM1 with thiostrepton induces cytotoxicity and enhances carboplatin sensitivity in cancer cells
Source: Oncotarget. 2014 Dec 4;5(22):11365–80. doi: 10.18632/oncotarget.2497 (PMC4294351; doi:10.18632/oncotarget.2497)
Supplement: Supplementary file 1 [file oncotarget-05-11365-s001.pdf]

SUPPLEMENTARY FIGURES AND TABLES

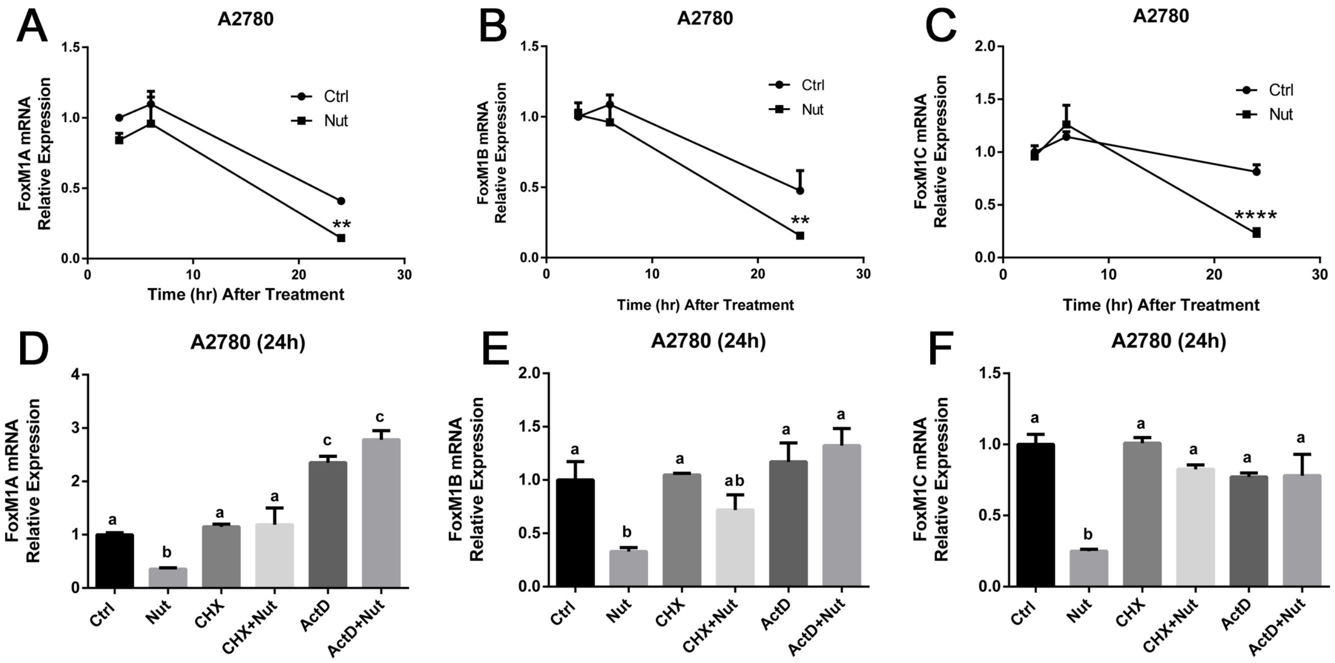

Supplementary Figure S1: Effects of Nutlin-3 on the expression of FoxM1 isoform A, B, and C in A2780 cells. Upper panel: Cells were treated with vehicle (0.05% DMSO) or 10 µM Nutlin-3 for 3, 6 or 24 h. Lower panel: Cells were treated with DMSO, Nutlin-3, CHX, CHX+Nutlin-3, ActD or ActD+Nutlin-3 for 24 h. Total RNA was isolated and subjected to real-time RT-PCR analysis. GAPDH was used for normalization of FoxM1 expression. Data are presented as Mean ± SD of 3 experiments. **indicates $P < 0.01$, and **** indicates $P < 0.0001$ by paired t-test. Difference alphabet letters indicate significant differences ($P < 0.05$) across treatments by One-way ANOVA.

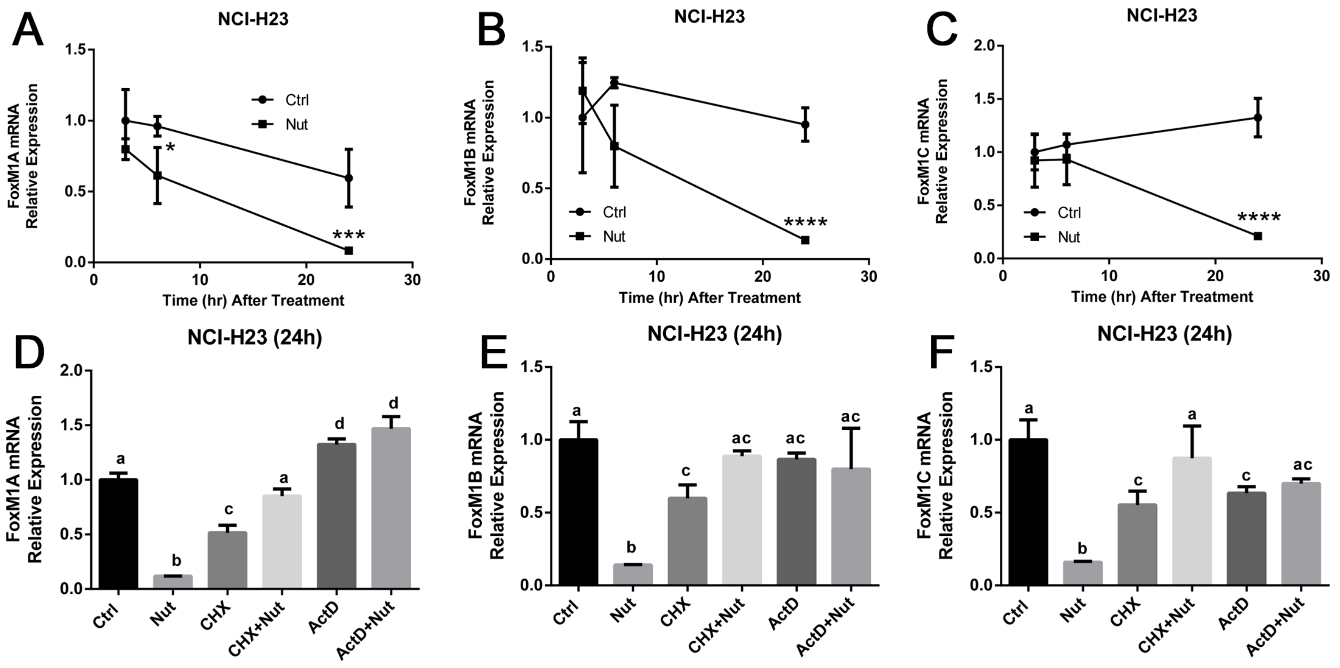

Supplementary Figure S2: Effects of Nutlin-3 on the expression of FoxM1 isoform A, B, and C in NCI-H23 cells. Upper panel: Cells were treated with vehicle (0.05% DMSO) or 10 µM Nutlin-3 for 3, 6 or 24 h. Lower panel: Cells were treated with DMSO, Nutlin-3, CHX, CHX+Nutlin-3, ActD or ActD+Nutlin-3 for 24 h. Total RNA was isolated and subjected to real-time RT-PCR analysis. GAPDH was used for normalization of FoxM1 expression. Data are presented as Mean ± SD of 3 experiments. *** indicates $P < 0.001$, and **** indicates $P < 0.0001$ by paired t-test. Difference alphabet letters indicate significant differences ($P < 0.05$) across treatments by One-Way ANOVA.

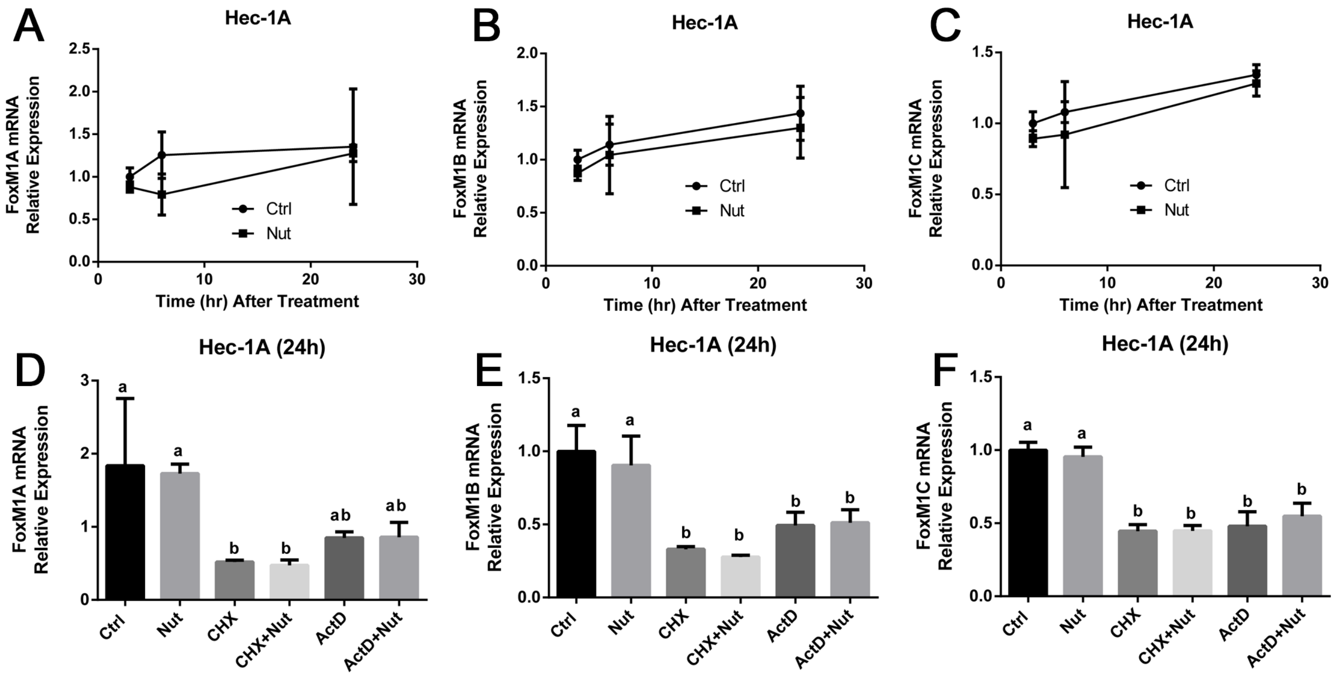

Supplementary Figure S3: Effects of Nutlin-3 on the expression of FoxM1 isoform A, B, and C in HEC-1A cells. Upper panel: Cells were treated with vehicle (0.05% DMSO) or 10 µM Nutlin-3 for 3, 6 or 24 h. Lower panel: Cells were treated with DMSO, Nutlin-3, CHX, CHX+Nutlin-3, ActD or ActD+Nutlin-3 for 24 h. Total RNA was isolated and subjected to real-time RT-PCR analysis. GAPDH was used for normalization of FoxM1 expression. Data are presented as Mean ± SD of 3 experiments. Difference alphabet letters indicate significant differences ($P < 0.05$) across treatments by One-way ANOVA.

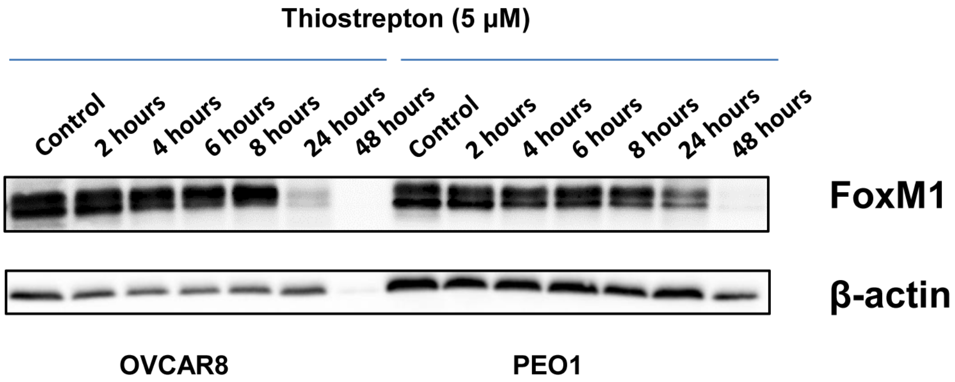

Supplementary Figure S4: Thiostrepton downregulates FoxM1 expression in OVCAR8 and PE01. Cells were treated with 5 µM Thiostrepton for indicated time points, and Western blot analysis was performed to determine FoxM1 expression. β-actin was used as the loading control. Down-regulation of β-actin at 48 h reflects proteolysis due to cell death.

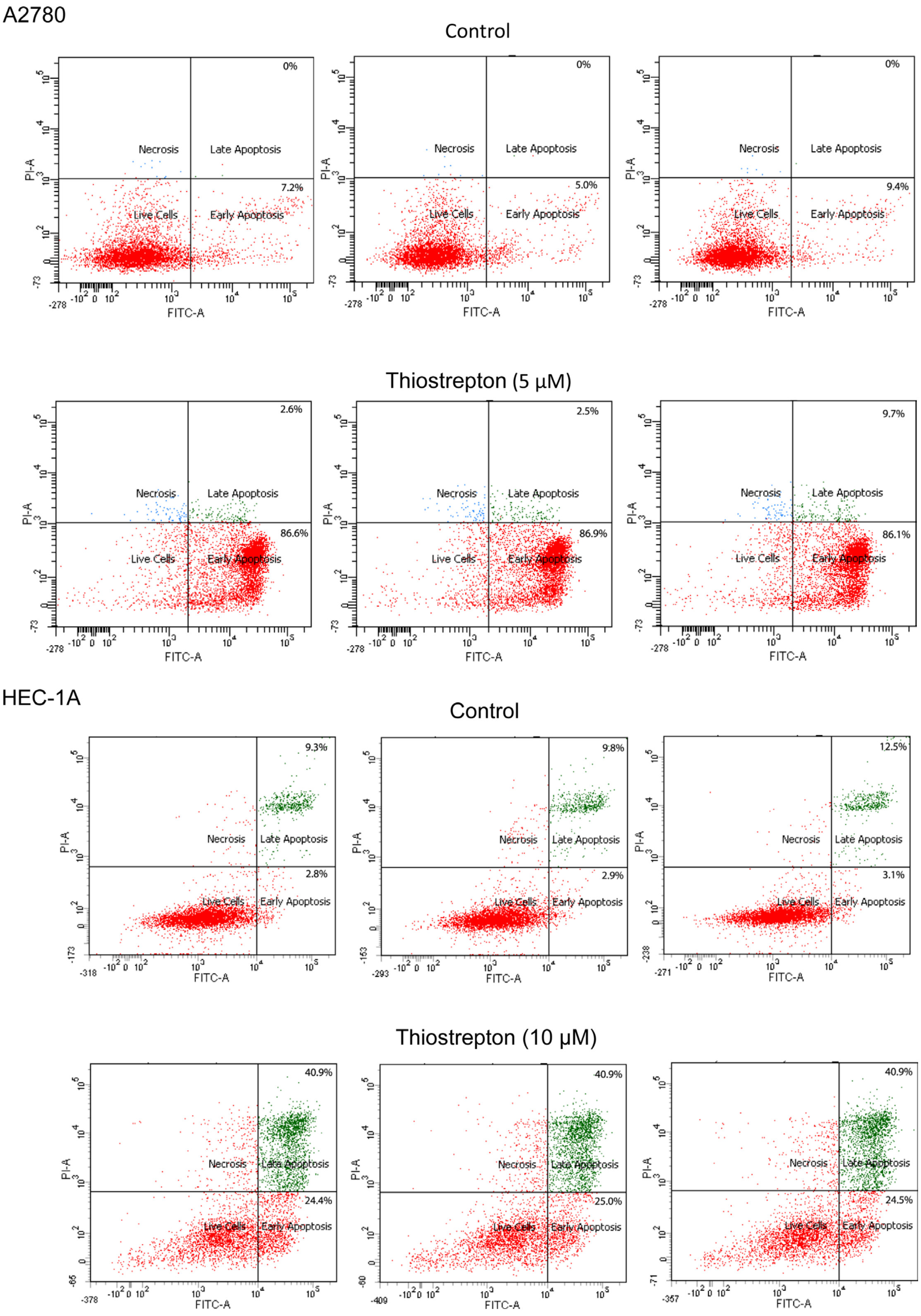

Supplementary Figure S5: Thiostrepton induces apoptosis in cancer cells. Cancer cells were treated with 5 µM (A2780) or 10 µM (HEC-1A) thiostrepton for 48 hours, and floating and attached cells were collected, stained with Annexin V (x-axis, FITC-A) and Propidium Iodide (PI) and sorted by flow cytometry. **(A)** Upper panels represent three replicates of untreated control cells, and low panels represents three replicates of A2780 cells treated with thiostrepton. Results indicate approximately 86% of cells undergoing early apoptosis compared to approximately 7.2% of cells undergoing early apoptosis in untreated cells. **(B)** In HEC-1A cells, both early and late apoptotic cells were observed in thiostrepton-treated cells.

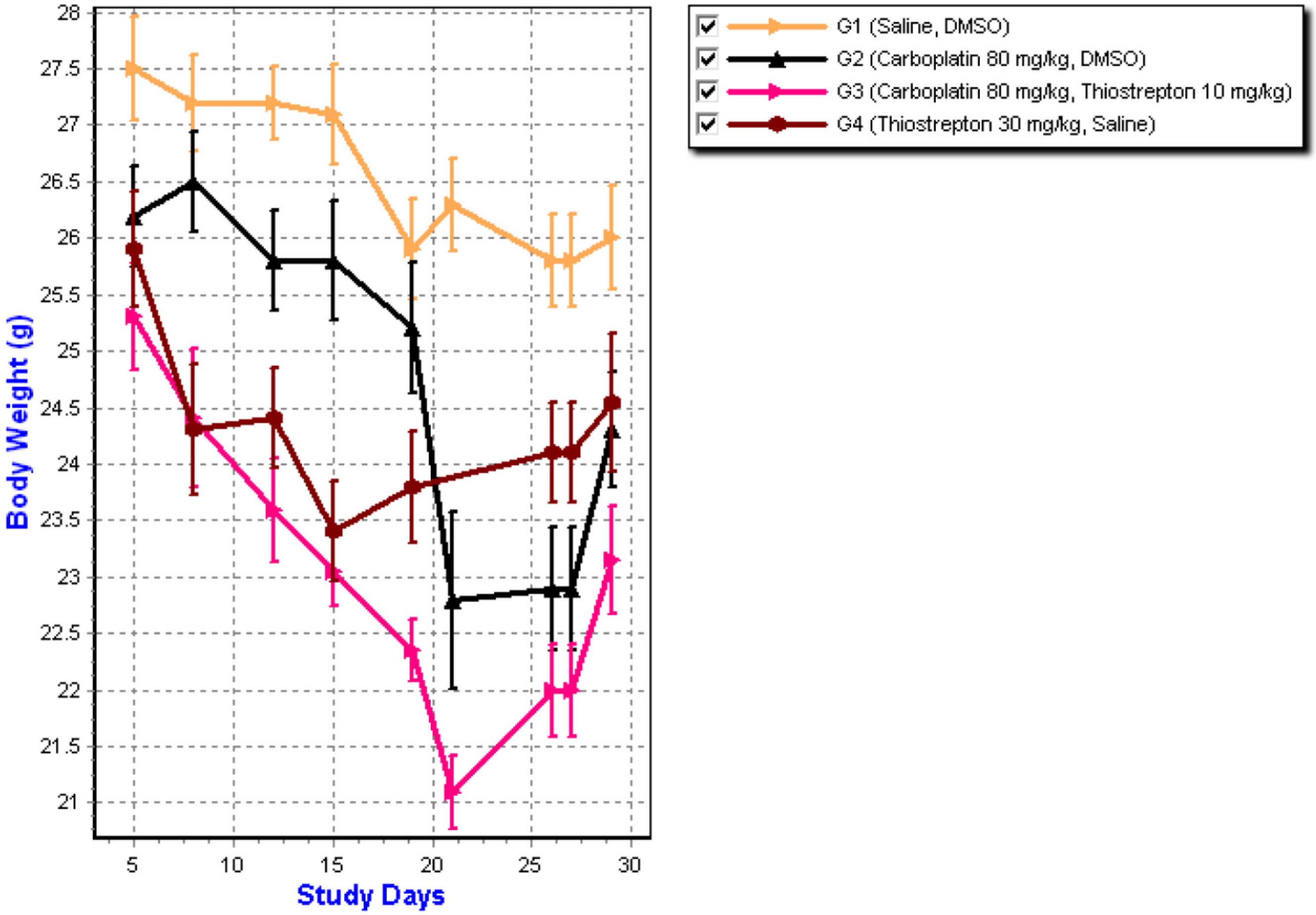

Supplementary Figure S6: Adverse effect of high dose of carboplatin. Dramatic weight loss was observed in mice treated with carboplatin alone (black line) or in combination with thiostrepton (red line) by day 21. Therefore, carboplatin dose was lowered to 20 mg/kg in subsequent treatments.

HEC-1A xenograft in DMSO-treated mouse

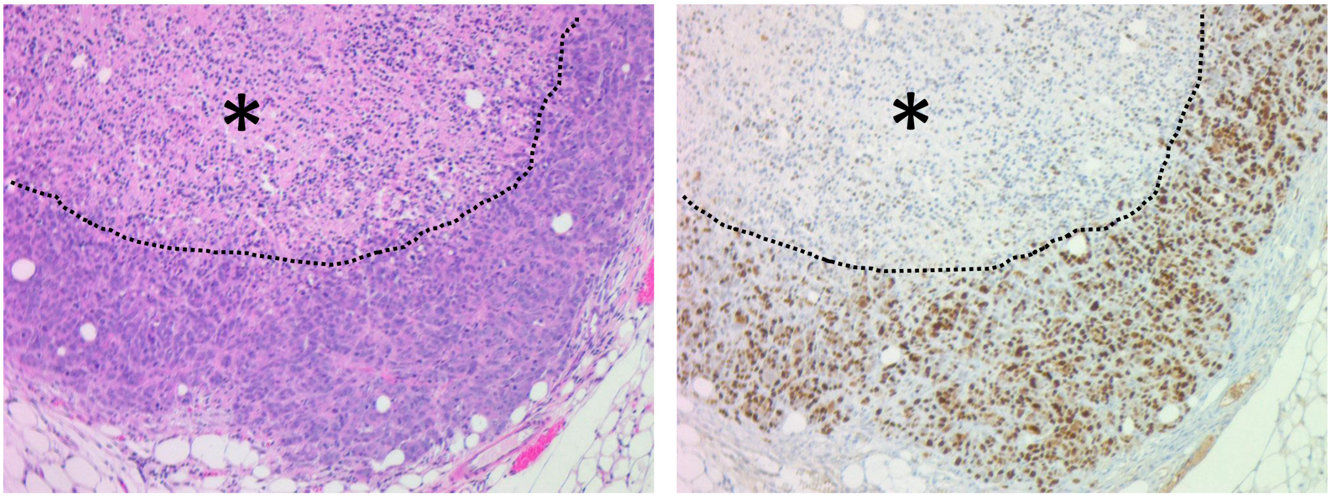

H&E p53

Supplementary Figure S7: Untreated HEC-1A tumor xenografts show large necrotic regions as indicated by Hematoxylin and Eosin (H&E) staining. Necrotic region containing pyknotic nuclei is indicated by black demarcation and asterisk. Serial section from the same region was stained with p53 antibody, and tumor cells surrounding the necrotic region show intense staining as expected because HEC-1A cells harbor mutant TP53.

Supplementary Table S1. Immunohistochemical analysis of FoxM1 expression

	FoxM1 - Cytoplasmic staining		FoxM1- Nuclear staining			
Patient ID	Intensity Score	% cells	Intensity Score	% cells	Grade	Histo-Subtype
1	2	100	2	90	III	Serous
2	2	100	2	90	III	Papillary serous
3	3	100	3	85	III	Papillary serous
4	3	100	3	85	III	Papillary serous
5	2	100	2	95	III	NOS - Adenocarcinoma
6	3	100	3	95	III	Papillary serous
7	3	100	3	85	III	Papillary serous
8	3	100	3	85	II	Papillary serous
9	3	100	3	85		Papillary serous
10	2	100	2	85	II	Papillary- mixed serous & mucinous
11	3	100	3	90	III	Carcinosarcoma
12	3	100	3	90	III	Papillary serous
13	3	100	3	95		Papillary serous
14	2 to 3	85% - 2, 15% - 3	3	85% - 2, 10% - 3	III	Clear cell
15	3	100	3	90	III	Papillary
16	3	100	3	90	III	Serous
17	3	100	3	90	III	Papillary serous
18	3	100	3	80	III	Serous and endometrioid features
19	3	100	2	80	III	Papillary, endometrioid, micropapillary, solid and clear patterns
20	3	100	3	80	III	Papillary serous
21	3	100	3	70	III	Serous
22	3	100	3	70	III	Papillary serous
23	3	100	3	80		Papillary serous
24	3	100	3	80	III	Papillary serous
25	3	100	3	80	III	Papillary serous
26	1	100	1	80	III	Papillary serous
27	3	20	3	75	III	Adenocarcinoma, Mixed type not otherwise specified
28	3	100	3	95	III	Serous (partial endometrioid components)
29	2	100	2	95	III	Mixed carcinoma, serous and transitional

(*Continued*)

	FoxM1 - Cytoplasmic staining		FoxM1- Nuclear staining			
**Patient ID**	**Intensity Score**	**% cells**	**Intensity Score**	**% cells**	**Grade**	**Histo-Subtype**
30	3	100	3	95	III	Mixed cell type with a predominant endometrioid and focal clear cell and papillary serous types
31	3	100	3	95	III	Papillary serous with psammoma bodies
32	3	100	3	95	II	Cystadenocarcinoma, mixed serous and endometrioid type
33	3	100	3	95	III	Serous with focal solid areas, endometrioid areas and transitional cell areas
34	3	95	3	80	III	Mixed papillary serous, solid and clear cell
35	3	100	3	75	III	Endometrioid carcinoma with focal clear, papillary, serous and squamous differentiation
36	2	100	2	75	III	Endometrioid and serous papillary features
37	3	100	3	80	III	Primary peritoneal adenocarcinoma, serous type
38	3	100	3	90	III	Papillary serous
39	3	100	3	95	III	Serous with psammoma bodies
40	2	100	2 to 3	60% - 2, 30% - 3	III	Papillary serous
41	3	100	3	90	III	Papillary serous
42	3	100	3	95	III	Papillary serous
43	3	100	3	95	III	Papillary serous
44	3	100	3	80	III	Predominate transitional cell with serous microglandular differentiation
45	3	100	3	95	III	Serous
46	3	100	3	95	III	Papillary serous
47	3	100	3	95	III	Serous
48	2	100	2 to 3	60% - 2, 30% - 3	III	Mixed mucinous and serous
49	3	100	3	95	II	Serous papillary cystadenocarcinoma

Supplementary Table S2. Real-Time RT-PCR primers

Gene	Forward	Reverse
FoxM1(total)	5′AGAATTGTCACCTGGAGCAG	5′TTCCTCTCAGTGCTGTTGATG
FoxM1A	5′GGTACACCCATCACCAGCTT	5′ATGGGTCTCGCTAAGTGTGG
FoxM1B	5′CGTGGATTGAGGACCACTTT	5′TCGGTCGTTTCTGCTGCTT
FoxM1C	5′CCCGAGCACTTGGAATCAC	5′TCCTCAGCTAGCAGCACCTT

Supplementary Table S3. p53 shRNA and FoxM1 shRNA sequences

shRNAs	Sequences
P53 shRNA-1 (V3LHS_333919)	TACACATGTAGTTGTAGTG
P53 shRNA-2 (V3LHS_333920)	TCTCTTCCTCTGTGCGCCG
FoxM1 shRNA-1 (V3LHS_396940)	AATAATCTTGATCCCAGCT
FoxM1 shRNA-5 (V2LHS_283849)	ATAATTAGAGGATAATTTG

Supplementary Table S4. Cell line STR genotypes

Cell Line	D5S818-Allele 1	D5S818-Allele 2	D13S317-Allele 1	D13S317-Allele 2	D7S820-Allele 1	D7S820-Allele 2	D16S539-Allele 1	D16S539-Allele 2	D16D539-Allele 3	vWA-Allele 1	vWA-Allele 2	TH01-Allele 1	TH01-Allele 2	TPOX-Allele 1	TPOX-Allele 2	CSF1PO-Allele 1	CSF1PO-Allele 2	D18S51-Allel 1	D18S51-Allel 2	D21S11-Allele 1	D21S11-Allele 2	D3S1358-Allele 1	D3S1358-Allele 2	D8S1179-Allele 1	D8S1179-Allele 2	FGA-Allele 1	FGA-Allele 2	Penta_D-Allele 1	Penta_D-Allele 2	Penta_E-Allele 1	Penta_E-Allele 2	AMEL
A2780	10	13	12	13	10		11	12	13	15	16	6		8	10	10		16	17	28	30	14	17	15	17	18	25	8		10		X
NCI-H23	10	13	12	13	10	10	11	12		15	16	6	6	8	10	10	10	16	18	28	30	14	16	15	17	19	23	8	9	10	13	X
HEC-1A	11		11		9	11	12			19	20	6	7	8	11	10				NA												X
ES2	11	13	11	11	11	11	11	13		16	17	9.3	9.3	8	12	10	15	13	16	32.2	33.2	15	18	14	14	21	21	8	13	13	16	X
SKOV3	11		8	11	14	14	12	12		17	17	9	9.3	11	11	11	11	16	17	31	31.2	14	14	14	15	24	25	12	13	5	13	X
PE01	11	12	10		10		9			15	16	9.3		9	11	10	12	16	17	32.2		16		13	13	20		14		12		X
OVCAR8	11	13	10		10		11			16		7	9.3	8	11	10		12		31		16		14		23		12		9	16	X
